# Cultural differences in motor skills and psychometric evaluation of the MABC-2 in Taiwanese school-aged children

**DOI:** 10.1038/s41598-025-25731-9

**Published:** 2025-11-07

**Authors:** Chia-Lin Koh, Tzu-Min Lee, Kuan-Lin Chen, Chien-Yu Huang

**Affiliations:** 1 Department of Occupational Therapy, College of Medicine, National Cheng Kung University, Tainan, Taiwan; 2 Department of Psychiatry, National Taiwan University Hospital, Taipei, Taiwan; 3 Department of Physical Medicine and Rehabilitation, College of Medicine, National Cheng Kung University Hospital, National Cheng Kung University, Tainan, Taiwan; 4 Institute of Allied Health Sciences, College of Medicine, National Cheng Kung University, Tainan, Taiwan; 5 School of Occupational Therapy, College of Medicine, National Taiwan University, No. 17, Xuzhou Rd., Zhongzheng Dist., Taipei City, 10005 Taiwan; 6 Department of Physical Medicine and Rehabilitation, College of Medicine, National Taiwan University Hospital, Taipei, Taiwan

**Keywords:** Movement assessment battery for children-2 (MABC-2), Cultural comparison, School-aged children, Psychometric properties, Motor skill, Neuroscience, Neurology

## Abstract

The Movement Assessment Battery for Children, Second Edition (MABC-2) is widely used to assess motor skills in school-aged children. This study aimed to evaluate the cultural differences in motor skills and psychometric properties of the MABC-2 in Taiwanese children. We assessed 257 children, with 71 assessed once, 168 assessed twice, and 90 completing a 6-month follow-up. We compared motor performance of Taiwanese with the standardized U.K. sample and examined the floor and ceiling effects, reliability, validity, and responsiveness of the MABC-2. The results showed that in general, Taiwanese children performed better in manual dexterity task but worse in aiming and catching tasks compared to the U.K. norm. The drawing trail task and balance tasks showed ceiling effects. Test-retest reliabilities were acceptable to high (ICC = 0.619–0.853), inter-rater reliabilities were high (ICC = 0.771–0.985), and random measurement errors were large (minimal detectable change = 33.9–171%). A three-factor model was identified for the subscales, and a one-factor model for the total scale. The MABC-2 showed weak to low correlations with the Vineland Adaptive Behavior Scales. Responsiveness ranged from small to large (effect size = 0.443–2.339). Accordingly, the MABC-2 demonstrated satisfactory psychometric properties, though local norms and cautious interpretation are needed.

## Introduction

Motor skills are essential for school-aged children to successfully perform daily activities, academic tasks, and sports within a school environment^1^. The Movement Assessment Battery for Children, Second Edition (MABC-2), is a globally recognized tool for assessing motor skills in this population^2,3^. It consists of eight tasks categorized into three domains: manual dexterity, aiming and catching, and balance^4^. Due to its brevity and engaging nature, the MABC-2 is widely used in both clinical and research settings as the gold standard to identify motor difficulties in children, and its normative data has been extensively used to identify children with developmental coordination disorder (DCD).

The MABC-2 was developed and normed in the U.K^4^. , but its applicability to school-aged children in Eastern cultures remains uncertain. In Taiwan, where no Chinese version of the MABC-2 is available, clinical practice relies on the UK norms from the original manual to convert raw scores into standard scores and assess motor skill deficits in children. However, motor skill development is profoundly influenced by social and cultural factors^5–7^, as children’s experiences with physical and manipulative activities may shape their motor abilities. Huang et al. found that preschool children in Taiwan outperformed their UK counterparts in manual dexterity and balance tasks, whereas UK children excelled in aiming and catching^5^. Similarly, Hua’s study of children in China reported comparable trends^8^. These findings suggest that lifestyle, educational practices, and cultural factors play a crucial role in shaping motor development. As a result, it is essential to investigate motor differences across different populations to ensure that assessments like the MABC-2 accurately reflect children’s motor abilities in children worldwide and provides valuable insights for researchers and clinicians in establishing reasonable expectations for children’s motor development.

Beyond comparing overall motor performance across cultures, it is also crucial to examine item-level performance on the MABC-2 to determine whether specific tasks are culturally appropriate. This includes investigating ceiling and floor effects, which can significantly impact the assessment tool’s sensitivity by limiting its ability to detect subtle motor difficulties or developmental progress^9,10^. When too many children score at either the maximum or minimum level, the test fails to capture meaningful individual differences, reducing its clinical and research utility. Huang et al.^5^ identified ceiling effects in three out of eight tasks when assessing preschool children in Taiwan, suggesting that certain items may not be sufficiently challenging for this population. Given the shared cultural background, similar effects may exist in school-aged children, yet there is currently little evidence in this age group. Without such data, the specific strengths and weaknesses of children in Eastern cultures remain unclear, increasing the risk that some tasks may fail to differentiate motor skills effectively, potentially leading to misclassification or inaccurate assessments. Investigating floor and ceiling effects in school-aged children is therefore essential for ensuring accurate identification of motor skill deficits across diverse populations. Such research would not only enhance the cross-cultural validity of the MABC-2 but also serve as a reference for refining the test to improve its applicability in Eastern cultures.

Furthermore, it is equally important to assess the psychometric properties of the MABC-2 when applied in different cultural settings. While the assessment’s reliability and validity have been extensively examined, there is a lack of evidence regarding its psychometric properties in school-aged children in Taiwan. Existing studies have primarily focused on preschool children or populations with DCD and intellectual disabilities in Taiwan and China^8,11^. For example, Hua et al. reported good internal consistency, strong inter-rater and test–retest reliability, and satisfactory construct validity in a large sample of preschool children^8^. Similarly, Wuang’s study on children with DCD demonstrated good internal consistency and excellent test–retest reliability^11^. However, psychometric properties are highly sample-dependent, and the reliability, validity, and responsiveness of the MABC-2 in school-aged children in Taiwan remain largely unknown. Conducting such evaluations is essential to ensure that the assessment produces accurate and meaningful results for this population.

As children transition into elementary school, the demands on their motor skills increase, making it crucial to understand their motor development and identify motor skill deficits among this period. Although the MABC-2 is widely used for motor skill assessment globally, its applicability and psychometric properties in Eastern cultures, particularly among school-aged children, remain largely unknown. This gap in knowledge limits the accurate interpretation of its results in these populations. Huang et al.^5^ previously used the MABC-2 to examine motor skill differences between Eastern and Western cultures; however, their study primarily focused on preschool children. Given the substantial differences in educational systems, parental expectations, and daily activities between preschoolers and school-aged children, performance trends may also vary between these age groups. Therefore, it is necessary to comprehensively investigate motor skill differences in school-aged children at both overall and item levels while also examining the psychometric properties of the MABC-2.

This study had two primary aims. First, it sought to evaluate both overall and item-level differences in motor performance between children in Taiwan and those in the U.K. Second, it aimed to examine the psychometric properties of the MABC-2, including test–retest and inter-rater reliability, construct validity, convergent validity, predictive validity, and responsiveness. We hypothesized that significant differences would be observed in certain domains and tasks of the MABC-2, underscoring the importance of considering cultural influences when adopting norms from other countries. Additionally, we expected the MABC-2 to demonstrate acceptable to excellent psychometric properties. The findings of this study will provide valuable insights into the cross-cultural applicability of the MABC-2, enhancing its cultural validity and ensuring its reliability and effectiveness in assessing motor skills across diverse populations.

## Methods

### Participants

Children in this study were recruited from various sources, including elementary schools, a rehabilitation clinic, and a youth developmental center in Taipei, New Taipei, Tainan, and Changhua. These locations are representative of western Taiwan, where the majority of the population resides. Inclusion criteria were children: (1) enrollment in elementary school (age 6 to 12 years), (2) absence of significant motor disabilities such as cerebral palsy, and (3) ability to follow at least two-step instructions. The exclusion criterion was parents who were unable to read Mandarin Chinese. Because the different age bands of the MABC-2 are applicable to children aged 3 to 6 (age band 1), 7 to 10 (age band 2) and 11 to 17 (age band 3) years, the children recruited in our study were further stratified into three age groups according to the different age bands of the MABC-2. This study received ethical approvals from National Taiwan University Hospital and E-DA Hospital.

### Measures

#### The movement assessment battery for Children, second edition (MABC-2)^4^

The MABC-2 is a motor skills assessment comprising 8 items grouped into 3 domains: manual dexterity, aiming and catching, and balance. Higher domain scores indicate better motor skills, and the sum of these domain scores provides an overall performance score. These summed scores are further converted into standard scores and percentiles. The mean of the standard score is 10, with SD of 3. The percentiles can be interpreted using a traffic light system. Percentiles equal to or lower than 5% indicate significant motor difficulties (red light), while those between 6% and 15% suggest borderline motor difficulties (yellow light). Percentiles above 15% suggest no significant motor difficulties (green light). The psychometric properties of the MABC-2 have been validated in several countries, ensuring its reliability and accuracy as an assessment tool^8,11–14^.

In this study, children administered the MABC-2 had their performance converted to standard scores on each domain and the total scale, based on the U.K. standardized sample. The standard scores were used to examine the floor and ceiling effects, as well as the psychometric properties of the MABC-2 and to compare the motor skills with the standardized sample from the U.K.

In addition, The MABC-2 checklist, a supplementary parent-report questionnaire, was used for examining the convergent validity of the MABC-2. The MABC-2 checklist assesses children’s performance in executing motor tasks in the school context. Each item is rated on a 4-point scale, with higher scores indicating worse motor performance.

#### The vineland adaptive behavior Scale, third edition (VABS-3)^15^

The VABS-3 was used as the criterion to assess the predictive validity of the MABC-2. It consists of three subscales: Caring for Self, Caring for Home, and Caring for Community. In this study, the Caring for Self and Caring for Home subscales were selected. Items within these subscales were rated on a 3-point scale. A higher sum score on the caring for self and caring for home subscales indicates a greater proficiency in adaptive behaviors related to self-care and domestic settings. The VABS-3 has good psychometric properties^15–17^.

### Procedures

Researchers responsible for data collection underwent rigorous training in administering the MABC-2. They were either occupational therapists or graduates of an occupational therapy program. As all graduates had completed internship training and had experience working with children with health conditions, they met the MABC-2 administration requirements.

Before data collection, researchers administering the MABC-2 received training from a senior occupational therapist (the corresponding author), who had extensive clinical experience with the MABC-2. They practiced administering the assessment with colleagues for at least two weeks, and their performance was evaluated at least twice. Researchers could begin data collection only after demonstrating smooth administration and achieving over 90% consensus in rating scores with other researchers. This training process ensured the quality and reliability of the evaluations.

To recruit participants for the study, we reached out to various sources, including school teachers, clinicians working in a rehabilitation clinic, and administrators at a youth development center. Facilities that expressed willingness to participate in the study were requested to distribute a cover letter explaining our research project and the informed consent forms to parents. Parents who agreed to have their children participate in the study returned the signed informed consent forms to the researchers. The assessment process involved three sessions for each child: the initial evaluation, a retest, and a follow-up at six months. During the initial evaluation, two researchers worked together to administer the MABC-2 assessment to each child. One researcher served as the main evaluator, while the other concurrently rated the child’s performance for inter-rater reliability. In addition to the MABC-2 assessment, parents were also asked to complete the MABC-2 checklist during the initial evaluation. The retest session occurred within the 2 weeks following the first session. Only the main evaluator at the first session administered the MABC-2. Six months later, at the follow-up session, parents were requested to fill out the caring for self and caring for home subscales of the VABS-3.

### Statistical analyses

The comparison of motor skills between children in Taiwan and the standardized sample from the U.K.

To compare motor skill differences across countries, we retrieved the raw mean scores and standard deviations for each item from the normative sample aged 6 to 10 years reported by Ke et al.^18^. Differences in motor skills for children aged 11 to 12 years were not analyzed due to the unavailability of raw mean scores and standard deviations, as well as insufficient sample sizes for these age groups. Tests of differences between two means using published summary statistics were conducted to compare the two samples.

#### The floor and ceiling effects of the MABC-2

The raw scores were examined using descriptive analyses. We examined the floor and ceiling effects for each item in different age groups (1 year as a group interval). More than 20% of the children achieving the highest or lowest raw scores on a particular item was indicative of a ceiling or flooring effect.

#### Reliability

Because Taiwan-specific normative data for the MABC-2 were unavailable, raw scores were transformed into age group–based z scores using the means and standard deviations of our sample within each age group, and were subsequently summed to generate domain scores and a total scale score for the examination of psychometric properties.

The intraclass correlation coefficient (ICC) _(2,1)_ was used to evaluate test–retest and inter-rater reliability, which is a two-way random-effects model with a single rating. ICC _(2,1)_ values greater than 0.9 were considered to indicate excellent reliability, while those exceeding 0.7 denoted good reliability, and values above 0.6 were considered acceptable for both test–retest and inter-rater reliability^18,19^.

To examine the magnitude of random measurement errors for the inter-rater and test–retest reliability of the MABC-2, we applied the standard scores to children in Taiwan and calculated the Minimal Detectable Change (MDC) along with its corresponding MDC percentage. The MDC is calculated by the following formula

$$\:\text{M}\text{D}\text{C}=1.96\times\:\text{S}\text{E}\text{M}\times\:\surd\:2.$$

Where SEM = $\:1\times\:\sqrt{(1-\text{r}\text{e}\text{l}\text{i}\text{a}\text{b}\text{i}\text{l}\text{i}\text{t}\text{y})},$ and reliability is test-retest or inter-rater reliability. For z-score, an MDC percentage above 100% indicates a notable presence of random measurement error in the assessment and thus potential sources of variability within the results^20–22^.

#### Validity

Convergent validity was examined with Pearson’s correlation coefficients (r) between the MABC-2 total standard score and the MABC-2 checklist total score. Moderate to high correlations indicate good convergent validity.

Predictive validity refers to the extent to which a test can accurately forecast future performance or outcomes on a related measure. It evaluates how well scores from one assessment correlate with later behavior, performance, or an established criterion. According to the Standards for Educational and Psychological Testing, the criterion variable is a measure of an attribute or outcome that is operationally distinct from the test itself. The test is not a direct measure of the criterion but rather a hypothesized predictor of that targeted outcome. Previous studies have shown an association between motor skills and children’s daily living skills in personal and domestic contexts, so the personal living and domestic living subscales was used as predictive criteria for the MABC-2. Predictive validity was examined with Pearson’s correlation coefficients (r) between the MABC-2 at initial evaluation and the caring for self and caring for home subscales of the VABS-3 at 6-month follow-up. Small to moderate correlations indicate good predictive validity^23^.

**Fig. 1:**
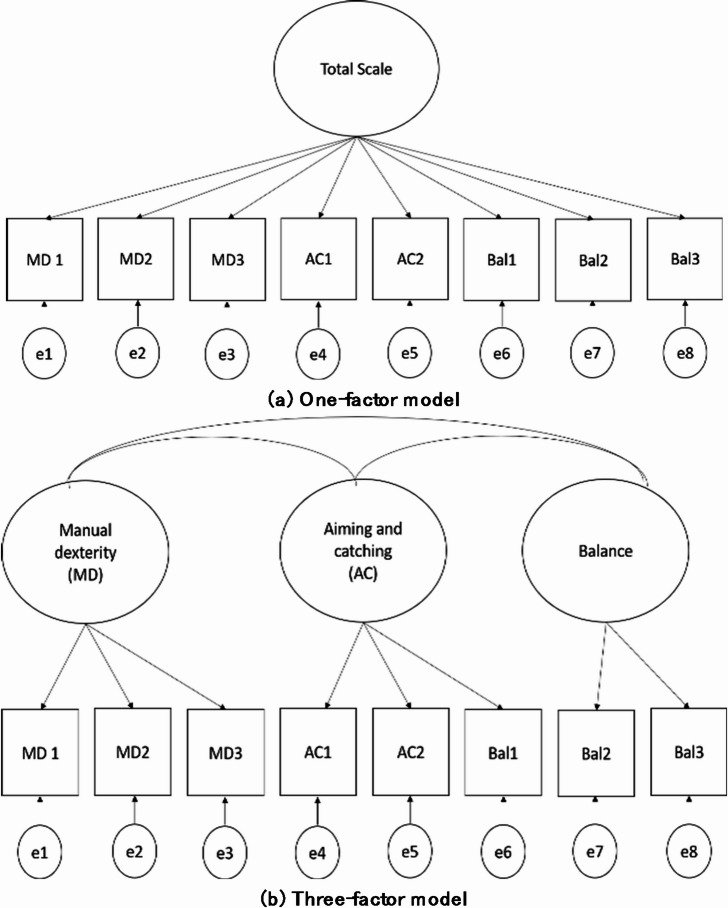
The factor structure of the MABC-2.

Construct validity was examined using confirmatory factor analyses. We employed two models to explore the factor structure of the MABC-2, as depicted in Fig. 1. The first model assessed whether the 8 tasks individually contributed to the 3 domains, while the second model investigated if the collective influence of the 8 tasks contributed to the overall motor skill performance. The two models helped to justify the appropriateness of using the domain and total scores of the MABC-2. The evaluation of model fit relied on the root mean square error of approximation (RMSEA), chi-squared, comparative fit index (CFI), Goodness-of-fit index (GFI), Adjusted goodness-of-fit (AGFI) and standardized root mean square residual (SRMR). Model performance was deemed satisfactory when meeting the criteria indicative of a well-fitting model: RMSEA < 0.06; CFI, GFI, and AGFI > 0.9; and SRMR < 0.05.

#### Responsiveness

Paired t-tests were used to investigate the difference in scores between the initial evaluation and the 6-month follow-up assessments. In addition, we calculated the effect sizes, with values of 0.2, 0.5, and 0.8 signifying small, moderate, and large responsiveness, respectively.

## Results

### Characteristic of participants

A sample of 257 children was recruited in our study, as detailed in Table 1. Of the total sample, 71 children received single assessments, 168 were assessed twice within 2 weeks, and 90 completed follow-up evaluations after a 6-month period. The majority of the children (76.4%) had no diagnosis of developmental disabilities. Table 1 also provides a breakdown of diagnoses among children with developmental disabilities, such as autism spectrum disorder and attention deficit hyperactivity disorder. Additionally, a significant proportion of parents demonstrated a high level of education, with 90.31% of mothers and 84.5% of fathers having completed education beyond the secondary stage.

**Table 1:** The demographics of the participants (*N* = 257).

Variables	Statistics
Age (year): mean (SD)	9.05 (1.74)
Sex (male): n (%)	147 (57)
Diagnosis: n (%)	
Autism spectrum disorder	16 (6.2)
Hyperactivity inattention disorder	30 (11.6)
Learning disabilities	7 (2.7)
Others (emotional disruption, hearing impairment, etc.)	9 (3.5)
Mother’s age: mean (SD)	38.28 (12.09)
Mother’s educational level: n (%)	
Up to junior high school	9 (3.49)
High school	54 (20.93)
Vocational School	41 (15.89)
College	109 (42.25)
Graduate school	34 (11.24)
Missing data	10 (4.26)
Father’s age: mean (SD)	40.30 (14.22)
Father’s educational level: n (%)	
Up to junior high school	15 (5.81)
High school	58 (22.48)
Vocational School	38 (14.73)
College	72 (27.91)
Graduate school	50 (19.38)
Missing data	24 (9.69)

### The comparison of motor skills between children in Taiwan and the standardized sample from the UK

Table 2 presents the mean raw scores for each item by boys and girls across the different age groups. Compared with the U.K. standardized sample, Taiwanese children generally performed better on manual dexterity tasks, particularly task 1 (i.e., placing coins/pegs) using the preferred hand, but performed worse on both aiming and catching tasks. In addition, within specific age bands, Taiwanese children showed better performance on balance task 1, namely standing on one foot.

**Table 2:** The comparison of motor skills for Taiwanese school-aged boys and girls with the standardized sample from the UK.

Items	6		7		8		9		10	
	Boys	Girls	Boys	Girls	Boys	Girls	Boys	Girls	Boys	Girls
MD^a^1-pref	17.33 (2.02)	16.06^*^ (2.05)	28.33^*^ (4.65)	28.07^*^ (3.73)	27.39 (5.11)	26.74 (3.88)	25.37 (5.50)	25.78 (4.79)	24.96 (4.85)	23.22^*^ (3.04)
MD1-npref	19.81 (3.18)	18.06 (3.11)	32.86^*^ (7.52)	31.2^*^ (5.2)	31.46 (6.63)	30.59 (5.09)	27.53 (5.24)	27.44 (5.46)	28.6 (5.32)	26.33 (2.95) ^*^
MD2	42.74 (9.84)	35.65 (7.84)	30.62 (7.79)	29.93 (6.85)	29.93 (19.17)	25.04 (6.69)	23.95^*^ (6.02)	21.22 (5.02)	23.32 (6.58)	21.11 (4.65)
MD3	0.85 (1.85)	0.00 (0.00)	0.95 (1.56)	0.80 (1.08)	0.43 (0.84)	0.48 (0.80)	0.26 (0.65)	0.33 (0.71)	0.48 (1.26)	0.06 (0.24)
AC^b^1	7.11^*^ (2.28)	7.35 (2.03)	3.71^*^ (3.33)	3.13^*^ (2.62)	6.21 (3.34)	4.78^*^ (2.55)	4.79^*^ (3.63)	3.22 (3.03)	5.60^*^ (3.95)	4.83 (3.26)
AC2	5.85 (2.38)	6.53 (1.66)	4.33^*^ (1.59)	5.13 (2.20)	5.36^*^ (2.66)	5.78 (1.70)	6.47 (1.84)	7.44 (1.13)	7.00^*^ (2.02)	6.61 (1.85)
Bal^c^1-Best	26.19^*^ (7.04)	28.29 (4.43)	17.10 (6.69)	19.33 (10.04)	21.54 (9.45)	22.67 (9.10)	24.58 (8.32)	24.44 (8.46)	23.80 (9.77)	27.61 (6.06)
Bal1-Other	20.26 (9.61)	25.29 (6.82)	9.29 (4.35)	10.80 (8.74)	15.18^*^ (10.42)	16.63 (10.53)	15.74 (10.26)	20.00 (10.00)	17.44 (10.96)	22.33^*^ (9.01)
Bal2	13.15 (3.67)	14.88 (0.49)	11.48 (3.98)	13.00 (4.05)	12.43 (4.41)	13.00 (4.05)	13.68 (2.91)	14.33 (1.32)	12.84 (3.80)	14.94 (0.24)
Bal3-best	4.22 (1.16)	4.65 (0.61)	4.38 (0.67)	4.73 (0.59)	4.57 (0.69)	4.56 (0.93)	4.63 (1.01)	4.78 (0.67)	4.80 (0.65)	4.89 (0.32)
Bal3-other	–	–	3.29 (1.15)	4.47 (0.74)	3.93 (1.33)	3.78^*^ (1.53)	4.37 (1.01)	4.44 (0.73)	4.32 (0.99)	4.50 (0.71)

^a^MD: Manual Dexterity,

^b^AC: Aiming and Catching. ^c^Bal: Balance

*Significant differences with the standardized sample from the UK (*p* < .05).

### The floor and ceiling effects of the MABC-2

Table 3 displays the results of the examination of floor and ceiling effects, but the results of the effects at age 12 were not further interpreted due to the limited sample size of only 7 children. Significant floor effects were observed in the task 1 of aiming and catching at ages 7 (22.4%) in the MABC-2. Significant ceiling effects (from 25.0% to 88.4%) were found in 4 of the 8 tasks across all age groups, including the drawing trail in the manual dexterity domain and all three tasks in the balance domain.

**Table 3:** The ceiling and floor effects of each item of the MABC-2^a^ in each age group.

Years	Effects	Manual dexterity				Aiming and catching			Balance				
		Task1_preferred hand n (%)	Task 1_nonpreferred hand n (%)	Task 2 n (%)	Task 3 n (%)	Task 1 Best hand n (%)	Task 1 Other hand n (%)	Task 2 n (%)	Task1_best foot n (%)	Task1_other foot n (%)	Task 2 n (%)	Task 3_ best leg n (%)	Task3_ other leg n (%)
6 (*n* = 44)	Ceiling	3 (6.8)	0 (0.0)	0 (0.0)	36 (81.8)	6 (13.6)	–	0 (0.0)	31 (70.5)	22 (50.0)	36 (81.8)	28 (63.6)	–
	Floor	0 (0.0)	1 (2.3)	0 (0.0)	3 (6.8)	2 (4.5)	–	0 (0.0)	0 (0.0)	0 (0.0)	2 (4.5)	3 (6.8)	–
7 (*n* = 36)	Ceiling	0 (0.0)	0 (0.0)	2 (5.6)	21 (58.3)	0 (0.0)	–	0 (0.0)	9 (25.0)	2 (5.6)	17 (47.2)	22 (61.1)	11 (30.6)
	Floor	0 (0.0)	0 (0.0)	1 (2.8)	1 (2.8)	8 (22.4)	–	1 (2.8)	0 (0.0)	0 (0.0)	0 (0.0)	0 (0.0)	0 (0.0)
8 (*n* = 55)	Ceiling	1 (1.8)	1 (1.8)	3 (5.5)	39 (70.9)	6 (10.9)	–	1 (1.8)	26 (47.3)	16 (29.1)	36 (65.5)	39 (70.9)	27 (49.1)
	Floor	2 (3.6)	2 (3.6)	1 (1.8)	0 (0.0)	2 (3.6)	–	7 (12.7)	1 (1.8)	2 (3.6)	0 (0.0)	6 (10.9)	2 (3.6)
9 (*n* = 28)	Ceiling	2 (7.1)	2 (7.1)	2 (7.1)	23 (82.1)	3 (10.7)	–	1 (3.6)	16 (57.1)	10 (35.7)	21 (75.0)	24 (85.7)	16 (57.1)
	Floor	1 (3.6)	0 (0.0)	0 (0.0)	0 (0.0)	4 (14.3)	–	0 (0.0)	0 (0.0)	0 (0.0)	2 (7.1)	1 (3.6)	0 (0.0)
10 (*n* = 43)	Ceiling	1 (2.3)	0 (0.0)	3 (7.0)	36 (83.7)	6 (14.0)	–	4 (9.3)	28 (65.1)	18 (41.9)	35 (81.4)	38 (88.4)	25 (58.1)
	Floor	1 (2.3)	2 (4.7)	0 (0.0)	1 (2.3)	6 (14.0)	–	1 (2.3)	2 (4.7)	2 (4.7)	2 (4.7)	5 (11.6)	0 (0.0)
11 (*n* = 44)	Ceiling	2 (4.5)	1 (2.3)	1 (2.3)	27 (61.4)	6 (13.6)	1 (2.3)	1 (2.3)	11 (25.0)	–	23 (52.3)	36 (81.8)	23 (52.3)
	Floor	1 (2.3)	0 (0.0)	1 (2.3)	0 (0.0)	3 (6.8)	9 (20.5)	1 (2.3)	0 (0.0)	–	1 (2.3)	8 (18.2)	3 (6.8)
12 (*n* = 7)	Ceiling	0 (0.0)	0 (0.0)	0 (0.0)	4 (57.1)	1 (14.3)	0 (0.0)	0 (0.0)	2 (28.6)	–	2 (28.6)	4 (57.1)	2 (28.6)
	Floor	0 (0.0)	0 (0.0)	0 (0.0)	0 (0.0)	1 (14.3)	2 (28.6)	2 (28.6)	0 (0.0)	–	2 (28.6)	3 (42.9)	1 (14.3)

^a^MABC-2: Movement Assessment Battery for Children, Second Edition.

### Reliability of the MABC-2

Tables 4 and 5 list the ICC values and MDC% of the three domains and the total scale. As for test–retest reliability, the ICC values were acceptable to high for the three domains and the overall performance (ICC = 0.619–0.853). Among the three domains, the reliability was relatively low in the domains of aiming and catching and balance (ICC = 0.619–0.764). The MDC% were large across the three age bands (106% to 171%).

**Table 4:** Test–retest reliability and random measurement error of the MABC-2.

Age band	Domain	Initial test mean (SD)	Retest mean (SD)	ICC^a^	MDC^b^ %
1	MD^c^	− 0.099 (1.918)	− 0.327 (1.660)	0.853	106
(*n* = 32)	AC^d^	− 0.107 (1.533)	− 0.047 (1.636)	0.619	171
	Balance	− 0.148 (2.062)	0.313 (1.619)	0.681	156
	Total	− 0.353 (4.465)	0.593 (3.866)	0.827	115
2	MD	0.215 (2.300)	0.012 (2.345)	0.781	130
(*n* = 99)	AC	0.133 (1.786)	0.233 (1.671)	0.691	154
	Balance	− 0.363 (2.292)	0.3061 (1.795)	0.682	156
	Total	− 0.446 (4.845)	0.527 (4.362)	0.812	120
3	MD	0.558 (2.092)	− 0.171 (2.168)	0.678	157
(*n* = 37)	AC	0.058 (1.596)	0.275 (1.667)	0.764	135
	Balance	− 0.168 (2.305)	0.118 (2.199)	0.800	124
	Total	− 0.414 (3.765)	0.836 (3.927)	0.822	117

^a^ICC: Intraclass Correlation Coefficient.

^b^MDC: minimal detectable change.

^c^MD: manual dexterity.

^d^AC: aiming and catching.

**Table 5:** Inter-rater reliability and random measurement error of the MABC-2.

Age band	Domain	Rater 1 mean (SD)	Rater 2 mean (SD)	ICC^a^	MDC^b^ %
1	MD^c^	− 0.592(1.489)	− 0.619 (1.504)	0.985	33.9
(*n* = 28)	AC^d^	0.939 (1.397)	0.835 (1.430)	0.913	81.7
	Balance	0.697 (1.600)	0.760 (1.598)	0.968	49.6
	Total	2.227 (3.645)	2.215 (3.747)	0.979	40.1
2	MD	0.023 (1.752)	0.309 (2.079)	0.928	74.3
(*n* = 51)	AC	0.014 (1.550)	− 0.139 (1.605)	0.963	53.3
	Balance	0.259 (2.203)	0.081 (2.284	0.979	40.1
	Total	0.415 (4.131)	− 0.217 (4.522)	0.964	52.6
3	MD	0.001 (1.639)	0.364 (1.972)	0.839	111
(*n* = 39)	AC	− 0.509 (2.495)	− 0.598 (2.514)	0.950	61.9
	Balance	− 0.943 (2.228)	− 1.328 (2.037)	0.771	133
	Total	− 1.453 (4.279)	− 2.290 (4.253)	0.868	101

^a^ICC: Intraclass Correlation Coefficient.

^b^MDC: minimal detectable change.

^c^MD: manual dexterity.

^d^AC: aiming and catching.

As for inter-rater reliability, the ICC values were generally high across the 3 age bands, ranging from 0.839 to 0.985, except the balance domain for Age band 3 (ICC = 0.771). The MDC% of the three domains and total scale were acceptable to large (MDC% =33.9–133).

### Validity of the MABC-2

In terms of convergent validity, small correlations were identified between the MABC-2 and the MABC-Checklist within age bands 2, with correlation coefficients − 0.302 (*p* = .001). No statistically significant correlations were observed in age band 1 and 3 (*p* > .05).

For construct validity, both the three-factor and one-factor models demonstrated adequate model fit. In the three-factor model, the manual dexterity domain was represented by three tasks, the aiming and catching domain by two tasks, and the balance domain by three tasks. After allowing a correlation between the error terms of Balance 2 and Balance 3, the model-fit indices were RMSEA = 0.058, CFI = 0.961, GFI = 0.972, AGFI = 0.938, and SRMR = 0.044. In the one-factor model, after allowing correlations between Task 1 and Task 2 of manual dexterity and between the two tasks of aiming and catching, all eight tasks loaded onto a single factor representing the total scale. The resulting model-fit indices were RMSEA = 0.052, CFI = 0.965, GFI = 0.973, AGFI = 0.946, and SRMR = 0.038.

As for predictive validity, only a small correlation was found between the total scale and the home subscales of theVABS-3 in age band 1 (*r* = .388, *p* < .05). Additionally, the moderate to high correlations between the MABC-2 checklist and the caring for self and caring for home subscales of the VABS-3 were significant in age bands 2 and 3 (*r* = -.491 to -0.864, *p* < .05).

### Responsiveness of the MABC-2

Table 6 displays the responsiveness of the MABC-2 across the three age bands. In general, for the three age groups, the differences in the mean standard scores on the three domains and total scale were significant (*p* < .05), except the domain of manual dexterity for age band 1 and 3. Moderate to large positive effect sizes (0.443–2.339) were found across the three age bands. Unexpectedly, negative trivial to small effect sizes were found for the subscale of aiming and catching in age bands 2 (-0.371) and 3 (-0.192).

**Table 6:** The responsiveness of the MABC-2.

Age band	Scale	Initial evaluation mean (SD)	6-month follow-up mean (SD)	Effect size	*p*-value
1 (*n* = 25)	MD^a^	− 0.192 (2.584)	0.729 (1.541)	0.921	0.069
	AC^b^	0.140 (1.747)	0.970 (1.375)	0.830	0.008
	Balance	− 0.221 (2.308)	0.965 (1.013)	1.186	0.004
	Total	− 0.273 (5.732)	1.206 (1.886)	1.479	0.003
2 (*n* = 56)	MD	− 0.451 (2.313)	0.375 (1.921)	0.826	< 0.001
	AC	− 0.029 (1.687)	− 0.400 (1.412)	− 0.371	< 0.001
	Balance	− 0.352 (2.171)	0.386 (1.769)	0.738	< 0.001
	Total	− 0.832 (4.430)	− 0.389 (2.706)	0.443	< 0.001
3 (*n* = 9)	MD	− 1.424 (1.822)	0.915 (1.414)	2.339	0.084
	AC	− 0.429 (1.803)	− 0.621 (1.758)	− 0.192	0.050
	Balance	− 0.366 (1.875)	1.350 (1.691)	1.716	0.013
	Total	− 2.218 (4.465)	− 0.186 (2.601)	2.032	< 0.001

^a^MD: Manual Dexterity.

^b^AC: Aiming and Catching.

All methods were carried out in accordance with relevant guidelines and regulations.

## Discussion

This study aimed to comprehensively evaluate the applicability of the MABC-2 from a measurement perspective in the context of Taiwan. Three key findings emerged from our investigation. First, notable ceiling effects were observed in the items related to the manual dexterity and balance domains. Second, the MABC-2 demonstrated satisfactory psychometric properties, including reliability, validity, and responsiveness. Third, distinct patterns of motor skills were evident among the children in Taiwan compared to the standardized sample from the U.K. These insights can aid clinicians in interpreting MABC-2 results more effectively when assessing school-aged children in Taiwan.

It is important to highlight that within the set of 8 tasks, significant ceiling effects were observed in 4 tasks in our Taiwanese sample. This suggests that these specific tasks might be relatively easy for children in Taiwan. The findings align with previous research indicating that Eastern children tend to excel in domains related to manual dexterity and balance^5^. Consequently, there is a potential task modification when utilizing the MABC-2 with children in Taiwan. For example, the paper sheet of the drawing trail task for age band 2 could be applied to age band 1. This insight underscores the need for future studies to delve deeper into this observation and its implications.

When comparing our sample’s performance to that of the standardized sample, we identified two key findings. First, children in Taiwan demonstrated superior manual dexterity. Second, their performance in aiming and catching was consistently lower across all three age bands.

The differences in motor performance can likely be attributed to two main factors. First, variations in motor skill development opportunities may play a role. Taiwanese children may have more exposure to activities that enhance manual dexterity but fewer opportunities to practice ball-handling skills^24^. Due to limited outdoor space, long school hours (8 AM to 5 PM), and additional cram school sessions, children in Taiwan often engage more in handwriting-related tasks than in ball sports. Moreover, although physical education classes include ball sports, they are not commonly practiced outside of school or in extracurricular activities, unlike in Western countries where free time for students is more prevalent. Second, as children progress through elementary school, they may increasingly participate in fine motor activities while reducing their involvement in gross motor tasks^25^. This shift in activity patterns may contribute to the observed motor performance differences. Overall, our findings highlight distinct motor skill development trends in Taiwanese children, emphasizing the need to establish culturally specific norms for motor performance in Taiwan.

The test–retest reliability was found to be acceptable. However, it was relatively low in the domains of balance and of aiming and catching across the three age bands of the MABC-2. These findings are consistent with previous research, which reported ICC values ranging from 0.23 to 0.97^26,27^. Two reasons may contribute to the relatively low test–retest reliability. First, a practice effect may be present in tasks in the balance domain. Upon review of the initial and retest mean standard scores on the balance domain, it was observed that the retest scores were consistently higher than the initial evaluation scores across all three age bands. This suggests that the children may have been more acquainted with the tasks during the second evaluation, resulting in improved performance and potentially affecting the test–retest reliability. Second, activities involving aiming and catching inherently entail unpredictable elements, such as the unpredictable trajectory of a thrown beanbag, which is challenging for children to control. Moreover, children in Taiwan may not be adept at such activities, as lower mean scores are evident in age bands 2 and 3. When children face challenges in ball-related activities, replicating their performance consistently at two different time points is difficult. Based on the insights gleaned from our study, we suggest that when administering the domains of balance and of aiming and catching, the practice opportunities outlined in the manual may not be sufficient for children; thus, providing additional practice opportunities may be advantageous in helping the children become familiar with the tasks. Increased familiarity could lead to performances that more accurately reflect their true abilities.

The inter-rater reliability of the MABC-2 demonstrated a high level of consistency, with ICCs ranging from 0.7 to 0.9. These results affirm that the rating instructions provided by the MABC-2 are clear and straightforward, facilitating uniformity in children’s performance assessment across different raters. Additionally, our training process uncovered valuable insights that can enhance rating accuracy. For instance, during the aiming and catching task, it is advisable for examinees to position themselves alongside the mats to mitigate angle-related issues with beanbag throws. Likewise, when observing children walking along a line, examiners should align their steps with those of the children, maintaining a position at the children’s side or back. As a result of these findings, it is evident that the MABC-2 can effectively assess school-aged children, regardless of the specific rater involved in the evaluation process.

It is important to note the large random measurement error of the MABC-2 across the three age bands, especially for test-retest reliability. Caution is warranted, as children’s performance may be largely influenced by random factors, meaning that only sufficiently large change scores can be interpreted as reflecting a true change in ability. This considerable level of measurement error may be explained by two factors. First, the MABC-2 includes only eight items, and a limited number of items can increase measurement error^28^. Second, while the tasks are reasonably balanced in terms of difficulty and effort required, this balance may contribute to variability in children’s performance. In addition, performance may also be influenced by chance factors.

The presence of considerable random measurement error suggests the need for more practice sessions to help children better understand the tasks and improve their proficiency. This is further supported by the increase in scores during the second evaluation, which may indicate that children possess the necessary abilities but may not fully demonstrate them initially due to task unfamiliarity. With additional practice, children become more comfortable with the tasks, enabling them to perform more consistently. Consequently, incorporating practice sessions could be a valuable strategy for minimizing the impact of random measurement errors on assessment results.

In our study, we have confirmed the presence of both three-factor and one-factor models for the MABC-2, and these models exhibit appropriate model-fit. This reaffirms the validity of the proposed models for the MABC-2. Previous research has generally supported the idea of a three-factor construct for the MABC-2, although some slight modifications may be necessary in certain cases^12–14^. Our study also introduced additional correlations the one-factor and the three-factor model was employed. Specifically, for the one-factor model, we found correlations between the first two tasks of the manual dexterity subscale and between two tasks related to the aiming and catching subscale. For the three-factor model, a correlation was added for the two tasks reflecting dynamic balance. These additional correlations can be justified by the shared fundamental components and similar execution methods inherent in these tasks. In summary, our results indicate that the eight tasks within the MABC-2 can be effectively grouped into three motor-related components or collectively represent an individual’s overall motor skill. In summary, these results also justify the assumption that applying the standard score of the U.K. norm did not affect the validity of the MABC-2. The subscale and total scores of the MABC-2 respectively represented the children’s fine motor, gross motor, and overall motor skills.

The correlations between the MABC-2 and the MABC Checklist showed small associations, and the correlation between the MABC-2 and the VABS-3 was only significant in age band 1. These findings suggest reasonable convergent validity and acceptable predictive validity. The presence of small correlations underscores the divergence in perspectives between caregivers and therapists, as well as the distinction between overall motor performance (the MABC checklist) and motor skills (the MABC-2). Notably, no predictive validity of the MABC-2 was observed in age bands 2 and 3. This phenomenon might be attributed to the fact that children in these age bands have likely reached a level of self-care proficiency where they have developed compensatory strategies for performing self-care tasks. Consequently, motor skill difficulties may not significantly impact their performance in self-care and household chore activities at this stage. However, it is conceivable that these motor skill challenges could influence more advanced skills required in school-related activities.

On the other hand, the MABC checklist exhibited strong correlations with the home and community scales of the VABS-3. The results suggested that motor performance had high correlations with skills in activities of daily living. The other plausible explanation was that both questionnaires were completed by parents.

In our study, we observed moderate to large responsiveness of the MABC-2. This finding aligns well with the natural developmental expectations for children, who are expected to develop their skills further over time. Consequently, the results indicate that the MABC-2 effectively captures and measures children’s progress, making it a valuable tool for assessing outcomes. However, it is important to note an unexpected discovery: negative effect sizes were identified in age bands 2 and 3 in the domain of aiming and catching. This unexpected outcome may be attributed to substantial random measurement error encountered during the baseline assessment, rendering the baseline results somewhat unstable. As a consequence, the follow-up tests were affected by this instability, leading to the observation of negative effects. These findings underscore a key point: when interpreting children’s performance on the domain of aiming and catching, a more conservative approach should be adopted, especially when a child’s performance lags behind that of their peers.

Our study sample was recruited from primary schools and a rehabilitation clinic across northern, central, and southwestern Taiwan, including children with and without diagnoses. These recruitment locations are representative of western Taiwan, where the majority of the population resides. This approach aligns with the sampling strategy of the MABC-2 standardization, which also included children with and without diagnoses. Additionally, approximately 5–10% of our sample comprised children with developmental conditions, consistent with their prevalence in the general Taiwanese population. The majority of our participants exhibited typical development, and those with diagnoses were enrolled in general education classes. Therefore, our findings can be considered generalizable to school-aged children in western Taiwan.

We identified two limitations in our study. First, the limited number of children in age band 3 who participated in the follow-up assessment might have constrained the representativeness of our sample. Second, when assessing construct validity (i.e., the factor structures of the MABC-2), we grouped all children together because the sample size was insufficient to conduct separate analyses for each age band. To enhance the robustness and validity of our findings, future studies should consider increasing the sample size.

## Conclusion

Motor skill development varies across cultures. Moreover, the MABC-2 demonstrated appropriate psychometric properties in our sample of school-aged children in Taiwan. The adequate psychometric properties suggest that the MABC-2 provides accurate and reliable assessments of children’s motor skills. However, two important considerations should be noted when using the MABC-2 with school-aged children in Taiwan. First, the presence of significant random measurement errors and performance variability should be considered, particularly in the aiming and catching domain. To address this, we recommend incorporating additional practice sessions or multiple assessments to improve the reliability of results in this specific domain.

## Data Availability

The data that support the findings of this study are available on request from the corresponding author, CYH, upon reasonable request.

## Declarations

### Impact statement

Overall, the MABC-2 proves to be an appropriate tool for assessing motor skills in school-aged children in Taiwan, when considering its measurement properties. Nevertheless, clinicians should exercise caution when interpreting the assessment results because of large random measurement errors in the domains of aiming and catching and balance, and different motor patterns existing in children in Taiwan.
